# Finding a chink in the armor: Update, limitations, and challenges toward successful antivirals against flaviviruses

**DOI:** 10.1371/journal.pntd.0010291

**Published:** 2022-04-28

**Authors:** Thamil Vaani Komarasamy, Nur Amelia Azreen Adnan, William James, Vinod RMT Balasubramaniam

**Affiliations:** 1 Infection and Immunity Research Strength, Jeffrey Cheah School of Medicine and Health Sciences, Monash University Malaysia, Bandar Sunway, Selangor, Malaysia; 2 Sir William Dunn School of Pathology, University of Oxford, Oxford, United Kingdom; Institut Pasteur, FRANCE

## Abstract

Flaviviruses have caused large epidemics and ongoing outbreaks for centuries. They are now distributed in every continent infecting up to millions of people annually and may emerge to cause future epidemics. Some of the viruses from this group cause severe illnesses ranging from hemorrhagic to neurological manifestations. Despite decades of research, there are currently no approved antiviral drugs against flaviviruses, urging for new strategies and antiviral targets. In recent years, integrated omics data-based drug repurposing paired with novel drug validation methodologies and appropriate animal models has substantially aided in the discovery of new antiviral medicines. Here, we aim to review the latest progress in the development of both new and repurposed (i) direct-acting antivirals; (ii) host-targeting antivirals; and (iii) multitarget antivirals against flaviviruses, which have been evaluated both in vitro and in vivo, with an emphasis on their targets and mechanisms. The search yielded 37 compounds that have been evaluated for their efficacy against flaviviruses in animal models; 20 of them are repurposed drugs, and the majority of them exhibit broad-spectrum antiviral activity. The review also highlighted the major limitations and challenges faced in the current in vitro and in vivo evaluations that hamper the development of successful antiviral drugs for flaviviruses. We provided an analysis of what can be learned from some of the approved antiviral drugs as well as drugs that failed clinical trials. Potent in vitro and in vivo antiviral efficacy alone does not warrant successful antiviral drugs; current gaps in studies need to be addressed to improve efficacy and safety in clinical trials.

## Introduction

Flaviviruses have been linked to human diseases since ancient times [1]. There are more than 70 different antigenically related viruses within the group that cause a wide spectrum of diseases in humans, ranging from hemorrhagic to neurological manifestations. Among the flaviviruses, Zika virus (ZIKV), dengue virus (DENV), Japanese encephalitis virus (JEV), West Nile virus (WNV), and yellow fever virus (YFV) have been regarded as important viruses due to their high morbidity and/or mortality [2,3]. In 2016, the World Health Organization (WHO) launched a research and development blueprint to accelerate the development of medical countermeasures for the world’s most dangerous pathogens [4]. In 2018, the list included “disease X,” which represents a serious international epidemic that could be caused by an unknown pathogen [5]. Experts have suggested that Coronavirus Disease 2019 (COVID-19), caused by Severe Acute Respiratory Syndrome Coronavirus 2 (SARS-CoV-2), can be considered the first disease X. However, another study regarded Zika as a disease X due to its new clinical syndromes, including microcephaly in newborns, which were not expected based on its history [6]. However, it is inevitable that there will be another disease X in the future with a potentially disastrous impact. Simpson and colleagues hypothesized that an emerging disease X would be a zoonotic virus, most likely a virulent RNA virus [7]. Among the zoonotic viruses, the mosquito-borne viruses, including flaviviruses, are on exponential growth due to climate change [8,9], urbanization [10,11], and increased international travel [12,13].

Despite extensive research and a wealth of information on the structural and biochemical properties of flaviviruses, currently, there are no specific antivirals available for any flavivirus infection. Vaccines are available only for JEV, YFV, and tick-borne encephalitis virus (TBEV). However, due to poor vaccination coverage and genotypic shift, the number of cases for these viruses remains quite high [14,15]. The world’s first approved dengue vaccine, tetravalent dengue vaccine CYD-TDV (Dengvaxia, Sanofi Pasteur, Lyon France), has been suspended in the Philippines due to its enhanced risk of severe dengue in seronegative individuals, a phenomenon associated with the occurrence of antibody-dependent enhancement (ADE) [16,17]. It was found that Dengvaxia elicited antibodies primarily specific to DENV4 [18], which supports the vaccine’s simulation of a primary monotypic infection. In addition, Dengvaxia elicited premembrane (prM)- and fusion loop epitope (FLE)-specific antibodies, both of which are effective ADE promoters [19]. Due to their increasing numbers, ongoing outbreaks worldwide, potential future epidemics, disease severity, global socioeconomic impact, and lack of treatment options for flaviviruses represent an urgent need for the development of effective antiviral agents. A large number of antiviral compounds have shown efficacy against flaviviruses in vitro. However, most of them have failed or have not been tested on animal models, and very few have proceeded into clinical trials [20–22]. In this review, we focus on both new and repurposed potential antiviral compounds for flaviviruses that have been evaluated both in vitro and in vivo.

### The current antiviral strategies

The traditional drug discovery pathway is complex, risky, lengthy, and expensive. The typical time taken to bring a drug from concept to the market is about 10 to 15 years and costs between $2.3 and $2.8 billion [23,24]. From 2015 to 2022, only 16 new drugs have obtained the Food and Drug Administration (FDA) approval for viral diseases, 5 of which are for the treatment of hepatitis C virus (HCV) and 5 for the treatment of human immunodeficiency virus (HIV) (www.fda.gov). Furthermore, for neglected tropical diseases, it is even more difficult because the financial returns may not cover the initial investment in research and development [25]. To address this scenario, drug repurposing strategy has recently gained increasing attention as a potential solution for rapid identification and development of new therapeutics [26,27].

The drug repurposing strategy uses existing approved or investigational drugs for new indications by exploring new molecular pathways and targets for intervention. It utilizes combinations of various databases, multiple omics technologies, and different evaluation methods [26,28,29]. One major advantage of the drug repurposing approach is that they can be fast-tracked through clinical Phase II as they have already passed regulatory hurdles and their pharmacokinetics, and safety have been established in preclinical models and early-stage clinical trials. This approach significantly reduces the time and cost of drug discovery for viral diseases. The recent COVID-19 pandemic highlighted the importance of drug repurposing in handling emerging outbreaks of viral diseases and the identification of promising candidates from drugs that had been forgotten [30]. Also, random screening of existing drugs or compounds for ZIKV resulted in the identification of potential candidates with anti-ZIKV activity in cells [31–33].

**Fig 1:**
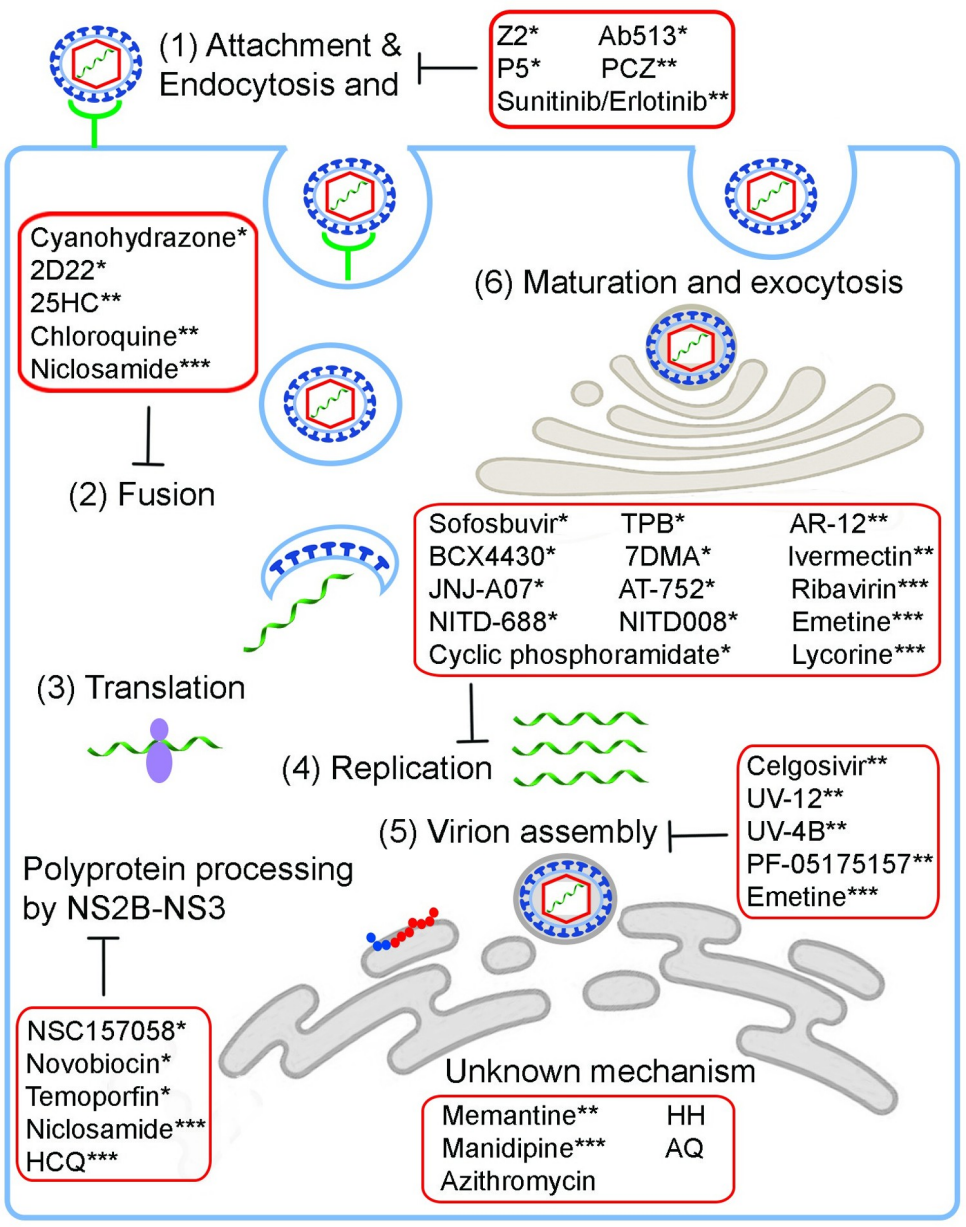
Drugs targeting the different steps in the flavivirus life cycle. Direct-acting antivirals*, host-targeting antivirals**, and multitarget antivirals*** that inhibit the different steps of the virus lifecycle discussed in this review are indicated in the red boxes. Flaviviruses consist of 3 structural proteins (C, prM or M, and E) and 7 NS proteins (NS1, NS2A, NS2B, NS3, NS4A, NS4B, and NS5), each playing distinctive roles in the different stages of the flavivirus life cycle and modulation of innate immune responses. During the virus replication cycle, the viral proteins interact with numerous host factors to modulate cellular pathways and induce infection. The flavivirus life cycle is outlined in Fig 1. (1) The E glycoprotein of the mature virion binds to the host cell membrane receptors and enters host cells via clathrin-mediated endocytosis [3]. (2) The acidic environment of the endosome triggers major structural changes in the E glycoprotein, inducing fusion of the host endosomal membrane with the viral E protein [38]. This is followed by the uncoating of the NC and the releasing of the viral genomic RNA into the cytoplasm. (3) The positive-sense viral genome (+ssRNA) is translated by ribosomes to form the viral polyprotein, which is then cleaved into structural and NS proteins by viral serine protease (NS2B-NS3) and host–cells peptidases/signalases in the ER [39]. (4) The NS proteins associate with intracellular membranes located on the surface of the ER to form viral replication complexes. The NS5 protein-derived RdRp synthesizes a complementary minus-strand RNA from genomic RNA for the synthesis of new positive-strand viral RNA [40]. (5) The newly synthesized viral RNA will then associate with the C protein in the ER to form a NC, leading to the formation of immature virions [41]. (6) Maturation and exocytosis. The assembled immature, noninfectious virion will then bud off the ER and travels through the secretory pathway to the TGN, where furin-mediated proteolysis occurs. Then, the mature virions are released from the cell surface via exocytosis [39]. C, capsid; E, envelope; ER, endoplasmic reticulum; M, membrane; NC, nucleocapsid; NS, nonstructural; prM, premembrane; RdRp, RNA-dependent RNA polymerase; TGN, *trans*-Golgi network.

Antiviral drugs can be divided into 3 groups based on their inhibitory mechanisms:

- **Direct-acting antivirals:** They directly or indirectly target the viral proteins to inhibit their biological functions in the virus life cycle (Table 1, Fig 1).
- **Host-targeting antivirals:** They target host proteins or pathways implicated in the different steps of the viral life cycle, immune system, and disease pathogenesis (Table 2, Fig 1). The advances in the understanding of virus–host interactions through genome-wide RNA interference (RNAi), CRISPR, and/or affinity purification mass spectrometry have led to the discovery of various cellular pathways and host factors [34,35].
- **Multitarget antivirals:** They are designed to simultaneously modulate multiple targets involved in the virus life cycle (Table 3, Fig 1) to achieve enhanced efficacy and/or improved safety [36,37]. They may offer a better pathway to develop successful antivirals against flaviviruses, which have complex molecular interactions between the virus and host cells.

**Table 1:** Direct-acting antivirals for flaviviruses.

Compound	Status/indication	Target	In vitro testing	Animal model (virus strain)	Regimen	Refs
Z2	-	E protein	ZIKV, DENV, and YFV	A129 mice (ZIKV GZ01) and AG6 mice (ZIKV SZ01)	10 mg/kg i.p. QD for 6 days from 1 hour pi	[42]
				Pregnant C57BL/6 mice (ZIKV SZ01)	10 mg/kg i.p. Single dose at 1 hour pi	
P5	-	E protein	JEV and ZIKV	BALB/c mice (JEV AT31)	1 μM i.p. Single dose along with virus infection	[43]
				AG6 mice (ZIKV H/PF)	50 μM i.p. Single dose along with virus infection	
Cyanohydrazone (JBJ-01-162-04)	-	E protein	DENV, ZIKV, and JEV	AG129 mice (Mouse-adapted DENV2 S221)	40 mg/kg i.p. BID for 3 days from 1 day prior to infection until day 3 pi	[45,46]
Ab513	-	E protein	DENV	AG129 mice (DENV2 NGC)	5 mg/kg or 25 mg/kg i.v. Single dose at 1 day prior to infection	[47]
				Humanized mice (DENV2 05K3295)	25 mg/kg i.v. Single dose at 1 day prior to infection or day 1 pi	
2D22	-	E protein	DENV	AG129 mice (DENV2 D2S10)	20 μg i.p. Single dose at 1 day prior to infection or day 1 pi	[48]
NSC157058	-	NS2B-NS3 protease	ZIKV	SJL mice (ZIKV PA259459)	30 mg/kg via drinking water QD for 5 days from day 1 pi	[49]
Novobiocin	Approved/antibiotic	NS2B-NS3 protease	ZIKV	Dexamethasone-immunosuppressed mice (ZIKV PRVABC59)	100 mg/kg s.c. Q12h from day 1–13 pi	[51,52]
Temoporfin	Approved/photosensitizer	NS2B-NS3 protease	ZIKV, DENV, WNV, JEV, and YFV	Immunocompetent BALB/c mice (ZIKV GZ01)	0.02 mg/mice i.p. QD for 2 days from day 0 pi	[32]
				A129 mice (ZIKV GZ01)	1 mg/kg i.p. QD for 5 days from day 0 pi	
JNJ-A07	-	NS4B	DENV	AG129 mice (DENV2 RL strain)	1, 3, 10, or 30 mg/kg p.o. BID for 3 days from 1 hour prior to infection 1, 3, 10, or 30 mg/kg p.o. BID for 6 days from 1 hour prior to infection 30 mg/kg p.o. BID for 6 days from day 1, 2, 3, 4, 5, or 6 pi	[54]
NITD-688	-	NS4B	DENV	AG129 mice (DENV2 TSV01)	10, 30, or 100 mg/kg p.o. BID for 3 days from 0 hour pi 30 mg/kg p.o. QD for 3 days from 0 hour pi	[55]
BCX4430 (Galidesivir)	Investigational/antiviral	RdRp	ZIKV, WNV, and TBEV	AG129 mice (ZIKV P 6–740)	150 mg/kg or 300 mg/kg i.m. BID for 8 days from 4 hours prior to infection 300 mg/kg i.m. BID for 7 days from day 1, 3, 5, or 7 pi	[61–64]
NITD008	-	RdRp	ZIKV	A129 mice (ZIKV GZ01)	50 mg/kg p.o. QD for 4 days from day 0 pi	[66]
				AG129 mice (DENV2 S221)	25 mg/kg p.o. BID for 3 days from day 0 or day 3 pi	
7DMA	-	RdRp	ZIKV and WNV	AG129 mice (ZIKV MR766)	50 mg/kg p.o. QD for 10 days from 1 hour prior to infection. 50 mg/kg p.o. QD for 5 days from 2 days prior infection	[68]
				BALB/c mice (WNV 13–104)	25 mg/kg i.p. BID for 19 days from day 0 pi or BID for 18 days from day 1 pi or BID for 16 days from day 3 pi or BID for 11 days from day 8 pi	[69]
Cyclic phosphoramidate (compound 17)	-		DENV	AG129 mice (DENV2 TSV01)	100 and 300 mg/kg, p.o. BID for 3 days from day 0 pi	[70]
AT-752	-		JEV, WNV, YFV, DENV, and ZIKV	AG129 mice (DENV2 D2Y98P)	1,000 mg/kg p.o. Single dose at 4 hours prior infection, followed by 500 mg/kg p.o. BID for 7 days from 1 hour pi	[72]
TPB	-	RdRp	ZIKV	BALB/c mice (ZIKV PRVABC59)	25 mg/kg i.p. 3 injections Q12h prior infection	[75]
Sofosbuvir	Approved/antiviral	RdRp	ZIKV	C57BL/6 mice (mouse-adapted ZIKV Dakar 41519)	33 mg/kg via drinking water QD for 7 days from day 1 pi	[76]
				NOD/SCID model (ZIKV ibH 30656)	50 mg/kg i.p. or p.o. QD for 10 days from day 1 pi	

BID, twice a day; DENV, dengue virus; i.g., intragastric; i.m., intramuscular; i.p., intraperitoneal; JEV, Japanese encephalitis virus; pi, postinfection; p.o., oral; Q12h, every 12 hours; QD, once a day; QID, 4 times a day; s.c., subcutaneous; TBEV, tick-borne encephalitis virus; TID, 3 times a day; YFV, yellow fever virus; WNV, West Nile virus; ZIKV, Zika virus.

**Table 2:** Host-targeting antivirals for flaviviruses.

Compound	Status/indication	Target	In vitro testing	Animal model (virus strain)	Regimen	Refs
PCZ	Approved/antipsychotic	D2R; clathrin	DENV and JEV	Stat1^−/−^ mice (mouse-adapted DENV2 NGC-N)	1 mg/kg and 5 mg/kg i.p. for the first treatment and p.o. for the following treatments BID for 10 days from 0 hour or 6 hours pi 4 mg/kg and 8 mg/kg i.p. 2-day intervals for 10 days from 0 hour or 6 hours pi	[80]
Sunitinib and erlotinib	Approved/ anticancer	AAK1 and GAK	DENV, ZIKV, and WNV	AGB6 and AG129 mice (N124D/K128E DENV2 PL046)	30 to 60 mg/kg, i.p. or p.o. QD for 5 days from 0 hour pi 30 to 60 mg/kg, i.p. or p.o. BID for 5 days from 0 hour pi	[81,82]
25HC	Investigational	Lipid metabolism	ZIKV, DENV, YFV, and WNV	BALB/c and A129 mice (ZIKV GZ01)	50 mg/kg i.p. QD for 7 days from 12 hours prior to infection	[83]
				NHP (ZIKV GZ01)	1.5 mg/kg i.v. QD for 7 days from 1 day prior to infection or 4 hours pi	
Chloroquine	Approved/antimalarial	Endosomal acidification	DENV and ZIKV	NHP (DENV2 NGC)	25 mg/kg p.o. single dose at 48 hours prior infection, followed by 25 mg/kg, p.o. QD for 4 days from day 1, 10 mg/kg p.o. QD for 4 days from day 5, 5 mg/kg p.o. QD for 2 days from day 9 25 mg/kg, p.o. QD for 4 days from day 1 pi, followed by 10 mg/kg p.o. QD for 4 days from day 5, 5 mg/kg p.o. QD for 2 days from day 9 25 mg/kg, p.o. BID for 4 days from day 1 pi, followed by 10 mg/kg p.o. BID for 4 days from day 5, 5 mg/kg p.o. BID for 2 days from day 9	[86]
				BALB/c mice and A129 mice (ZIKV GZ01)	100 mg/kg i.g. QD for 5 days from 6 hours prior to infection	[88]
Ivermectin	Approved/antiparasitic	IMPα/β1	ZIKV, DENV, and WNV	Ifnar1^−/−^ (ZIKV Senegal)	4 mg/kg i.p, 4 doses (at 2 days prior infection, day 0, 2 and 4 pi)	[94,95,97]
AR-12 (P12-34)	Investigational/anticancer	PI3/AKT pathway and GRP78; DHODH	DENV, ZIKV, and JEV	ICR suckling mice (DENV2 PL046)	25 mg/kg i.c. and i.p. Single dose at 0 hour, 12 hours or 24 hours pi	[92,93]
				Stat1^−/−^ mice (mouse-adapted DENV2 NGC)	2.5 mg/kg i.p. QD for 5 days from day 0 pi	
Celgosivir	Investigational/antiviral	ER α-glucosidases I	DENV	AG129 mice (clinical isolates of DENV1 and DENV2, and mouse-adapted DENV2 S221)	50 mg/kg p.o. BID for 3 days from day 0 or 3 pi 50 mg/kg p.o. QID until day 6 pi from day2 or 3 pi	[67]
UV-12	-	ER α-glucosidases I and II	DENV	AG129 mice (mouse-adapted DENV2 S221)	20 to 100 mg/kg p.o. TID for 7 days from 1 hour prior to infection	[101]
UV-4B	-	ER α-glucosidases I and II	DENV	AG129 mice (mouse-adapted DENV2 S221)	10 to 100 mg/kg p.o. TID for 7 days from 1 hour prior to infection or day 1 or 2 pi	[100]
PF-05175157	Investigational/antidiabetic	ACC	WNV, ZIKV, and DENV	Swiss albino CD-1 mice (WNV NY99)	20 mg/kg p.o. BID for 7 days from 1 day prior to infection	[102]
				C57BL/6 ACC2^-/-^ mice (WNV NY99)	20 mg/kg p.o. BID for 7 days from 1 day prior infection	
Memantine	Approved/Alzheimer disease	NMDAR	ZIKV	C57BL6j WT mice (ZIKV HS-2015-BA-01)	30 mg/kg p.o. BID from day 2 to 6 pi	[103]

25HC, 25-hydroxycholesterol; ACC, acetyl-coenzyme A carboxylase; BID, twice a day; DENV, dengue virus; i.g., intragastric; i.p., intraperitoneal; JEV, Japanese encephalitis virus; PCZ, prochlorperazine; pi, postinfection; p.o., oral; QD, once a day; QID, 4 times a day; s.c., subcutaneous; TID, 3 times a day; WNV, West Nile virus; WT, wild-type; YFV, yellow fever virus; ZIKV, Zika virus.

**Table 3:** Antiviral with multiple mechanisms of action.

Compound	Status/indication	Flavivirus target	In vitro testing	Animal model (virus strain)	Regimen	Refs
Niclosamide	Approved/antihelminthic	NS2B-NS3 protease; Endosomal acidification	DENV, ZIKV, WNV, JEV, and YFV	ICR suckling mice (DENV2 PL046)	2 to 5 mg/kg i.p. Single dose at 0 hour pi	[32,104,105]
Ribavirin	Approved/antiviral	RdRp; host immune response	ZIKV and DENV	Stat1^−/−^ (ZIKV MR 766)	15 mg i.p. QD for 3 days from day 0 pi 10 mg i.p. QD for 9 days from day 0 pi	[106,116]
Emetine	Approved/antiparasitic	RdRp; lysosomal function	ZIKV	Ifnar1^−/−^ mice (ZIKV FSS13025)	1 to 2 mg/kg i.p. QD for 3 days starting from 24 hours prior to infection or day 1 pi	[109]
				Immunocompetent SJL mice (Brazil-ZIKV2015)	1 mg/kg i.p. QD for 6 days from day 0 pi	
Lycorine	-	RdRp; HSP70	ZIKV	AG6 mice (ZIKV SZ-WIV001 KU963796)	1, 5, or 10 mg/kg, i.g. QD for 14 days from day 0 pi	[112]
Manidipine	Approved/antihypertensive	Calcium channel; NS4B	JEV, ZIKV, DENV, and WNV	BALB/c mice (JEV AT31)	25 mg/kg i.p. BID for first 2 days and QD for the next 19 days	[113]
HCQ	Approved/antimalarial	Autophagy; NS2B-NS3 protease	ZIKV	WT pregnant mice (Brazil-ZIKV2015)	40 mg/kg i.p. QD for 5 days from day 1 pi.	[114,115]

BID, twice a day; DENV, dengue virus; HCQ, hydroxychloroquine; i.g., intragastric; i.p., intraperitoneal; JEV, Japanese encephalitis virus; pi, postinfection; p.o., oral; QD, once a day; QID, 4 times a day; s.c., subcutaneous; TID, 3 times a day; YFV, yellow fever virus; WNV, West Nile virus; ZIKV, Zika virus.

### Direct-acting antivirals

#### Envelope protein inhibitors

A synthetic peptide derived from the conserved stem region of the ZIKV E protein termed Z2 was designed to target the E protein. Flow cytometry analysis revealed the binding of Z2 with the ZIKV E protein expressed on cells. Z2 exhibited strong inhibitory activity against ZIKV, DENV, and YFV in vitro. In ZIKV-infected A129 mice, intraperitoneal (i.p.) administration of Z2 at 10 mg/kg once a day (QD) for 6 days reduced the viral load, prolonged the mean survival of the mice, protected 75% of the mice from death, and, importantly, no neurological symptoms were observed in the treated mice. The efficacy of Z2 was also observed in AG6 mice, in which Z2 reduced the viral load by 13-fold, prolonged the survival of mice, and conferred 67% protection from death. Importantly, Z2 reduced viremia in infected pregnant C57BL/6 mice and reduced the viral load in placentas and fetal heads, indicating protection against vertical transmission. The peptide was shown to be safe for pregnant mice and fetuses up to 120 mg/kg [42].

Another peptide, P5 derived from the helix 2 of the JEV E protein stem, demonstrated inhibitory activity against JEV and ZIKV in vitro. In the JEV-infected BALB/c mice, treatment with a single dose of 1 μM of P5 via i.p. resulted in 67% of survival, while mice in the control group exhibited only 20% of survival. Treatment with P5 also reduced the viral load and inflammation in the JEV-infected mouse brain. For ZIKV, treatment of infected AG6 mice with a single dose of 50 μM of P5 via i.p. reduced the histopathological damage in the brains and testes [43]. The exact mechanism of the P5 peptide was not explored in this study. However, helix 2 peptides have been proposed to block virus infection through a nonspecific and hydrophobic membrane binding step, which is followed by interaction with the E protein during the fusion step [44].

Cyanohydrazone, a class of DENV E inhibitors, was identified through a medicinal chemistry approach. The compound exhibited significant antiviral activity against DENV, ZIKV, and JEV in cell culture. A refined cyanohydrazone compound, JBJ-01-162-04, exhibited improved metabolic stability and toxicity profile. Prophylactic treatment (1 day prior to virus infection) of DENV2-infected AG129 mice with 40 mg/kg of JBJ-01-162-04 twice a day (BID) for 3 days via i.p. reduced viremia [45]. A liposome-based in vitro assay demonstrated that cyanohydrazone prevented E-mediated membrane fusion by interacting with a conserved pocket n-octyl-β-D-glucoside (βOG) on the E protein [45,46].

Furthermore, because neutralizing antibodies primarily target viral E protein, monoclonal antibodies (mAb) inhibiting viral entry have been actively investigated as a potential antiviral. In this context, a therapeutic antibody called Ab513, which specifically binds to envelope domain III (EDIII) of the DENV, was developed through structure-guided methods. X-ray co-crystal structures confirmed the binding of Ab513 to the A-strand epitope on DENV4 EDIII with 20 H-bonds and 13 salt bridge interactions found across the Ab513-EDIII interface. Ab513 has been shown to possess high-affinity binding to multiple genotypes within all 4 serotypes and effectively neutralizes them. A single prophylactic administration (1 day prior to DENV2 injection) of Ab513 at 5 mg/kg and 25 mg/kg via the intravenous (i.v.) route in AG129 mice resulted in a 1.7 log10 and 2.4 log10 reduction in viral titer, respectively. Treatment with Ab513 also improved the survival of infected mice and conferred protection against central nervous system (CNS)-related symptoms, including paralysis. Administration of Ab513 (25 mg/kg) prior to or postinfection (pi) in humanized mice resulted in the recovery of human platelet levels, indicating the ability of Ab513 to prevent thrombocytopenia. In addition, treatment with Ab513 pi reduced vascular leakage in key organs. Importantly, Ab513 still retains its protective efficacy against DENV in the presence of heterologous enhancing antibodies. Ab513 prevented disease enhancement in DENV2 infected pups born to DENV1 immune mothers [47].

Another group developed 2D22, a human mAb specific to DENV2. In AG129 mice, the administration of a single dose of 2D22 (20 μg) at 1 day prior to DENV2 infection reduced the viral load in serum and bone marrow. Administration of 2D22-LALA at 1 day pi in a high-dose DENV2 lethal challenge model protected mice from death, while mice in the control group experienced nearly 90% fatality. Treatment of the ADE DENV2 lethal challenge model (AG129 mice pretreated with DENV1 serum followed by infection with DENV2) with 2D22 prevented the development of antibody-enhanced lethal vascular leakage. Cryo-electron microscopy (cryo-EM) revealed that 2D22 binds to all 3 domains of the E protein and locks both ends of all dimers on the virus surface, preventing glycoprotein E reorganization, which is required for virus fusion [48].

#### NS2B-NS3 protease inhibitors

An allosteric small-molecule inhibitor, NSC157058 was found to significantly inhibit ZIKV NS2B-NS3pro in vitro [49]. This study proposed that NSC157058 is likely to interfere with the folding of the NS2B cofactor based on the modeling of the compound bound to ZIKV NS2B-NS3pro [49], and another study demonstrated the binding of NSC157058 to WNV NS3pro exosite [50]. NSC157058 inhibited ZIKV infection in human fetal neural precursor cells (hfNPCs). In SJL mice, administration of NSC157058 via drinking water at 30 mg/kg QD for 5 days after 1 day pi significantly reduced the levels of circulating ZIKV about 10-fold compared to the untreated control mice [49].

Novobiocin, an antibiotic, was found to attach to the binding pocket of ZIKV NS2B-NS3 and causes inhibition of NS2B-NS3 protease activity. The ability of novobiocin to inhibit ZIKV NS2B-NS3 protease activity was further confirmed through a fluorescence-based protease inhibition assay [51]. Novobiocin has shown strong inhibitory activity against ZIKV and DENV in cell culture [51,52]. Treatment of ZIKV-infected dexamethasone-immunosuppressed mice with novobiocin at 100 mg/kg every 12 hours (Q12h) from 1 to 13 days pi via the subcutaneous (s.c.) route resulted in 100% survival and a significant decrease in viral load in blood and organ tissues [51]. However, it is important to note that novobiocin, which was initially approved for the treatment of *Staphylococcus aureus*, has been withdrawn from the United States market due to its unfavorable pharmacological properties and safety profile [53].

High-throughput screening (HTS) of FDA-approved drugs led to the identification of another flavivirus NS2B-NS3pro inhibitor, temoporfin. Structural docking revealed the binding of the drug to the NS3 pockets into which NS2B binds and subsequently inhibits flavivirus polyprotein processing. In addition, temoporfin inhibited NS2B-NS3 protease activity in a protease inhibition assay. Preincubation of temoporfin with immobilized GST-NS3 beads significantly reduced the binding of the FLAG-NS2B to the GST-NS3, indicating the ability of the drug to disrupt the interactions between NS2B co-factor and the NS3 protease domain.

The binding of temoporfin was also further confirmed by protein thermal shift assays and surface plasmon resonance (SPR). The compound inhibited ZIKV infection in human placental cells and human neural progenitor cells (hNPCs). It also showed inhibitory activity against other flaviviruses, including DENV, WNV, JEV, and YFV in vitro. In a viremia immunocompetent BALB/c mouse model, treatment with temoporfin at 0.02 mg/mice QD for 2 days via i.p. reduced ZIKV RNA about 100-fold compared to the control group. On the other hand, in a lethal A129 mouse model, treatment at 1 mg/kg QD for 5 days protected 83% of the ZIKV-infected mice and prevented neurological symptoms such as hind limb weakness and paralysis [32].

#### NS4B inhibitor

JNJ-A07, a highly potent DENV inhibitor was identified through a large-scale cell-based anti-DENV2 screen. It exhibited antiviral potency against a panel of 21 clinical isolates, which included genotypes from all 4 serotypes in nanomolar (nM) to picomolar (pM) concentrations. Notably, the compound did not show antiviral activity against other RNA and DNA viruses. Resistance selection and reverse genetics studies used in this study identified NS4B as the molecular target for JNJ-A07. It was further identified that the compound prevented the NS3–NS4B interaction by causing a conformational change in the cytosolic loop of NS4B [54]. In DENV-infected AG129 mice, prophylactic treatment with JNJ-A07 via the oral (p.o.) route 1 hour prior to infection effectively reduced viral loads and virus-induced disease at a concertation as low as 3 mg/kg in both lethal and nonlethal DENV challenge models. Importantly, its antiviral potency was maintained even when treatment started several days after infection [54].

NITD-688 showed strong potency against all DENV serotypes with half-maximal effective concentration (EC_50_) values between 8 and 38 nm. However, the compound exhibited a higher EC_50_ value for CHIKV (> 20 μM) and other flaviviruses (>9.4 μM), demonstrating its specificity against DENV. Treatment with 30 mg/kg and 100 mg/kg BID for 3 days reduced viremia by 1.44 and 3.01 log, respectively [55]. When administered at 48 hours pi at 30 mg/kg BID, the compound resulted in a 1.16 log reduction in viremia on day 5. Selection of resistance mutation studies revealed NS4B as the target of NITD-688. The EC_50_ values of NITD-688 increased dramatically for NS4B mutant DENV2 replicons. This was further confirmed through nuclear magnetic resonance (NMR) spectroscopy studies, which demonstrated direct binding of NITD-688 to the viral protein NS4B. In addition, NITD-688 did not bind to NS4B with mutations T215A and A222V. Pharmacokinetic studies in rats and dogs revealed that the drug has a long elimination half-life and good oral bioavailability. In exploratory toxicology studies, the drug was well tolerated after 7-day repeat oral dosing [55].

#### NS5 RNA-dependent RNA polymerase inhibitors

Numerous nucleoside analogs that target viral polymerases have been widely used to treat several viral infections. Their mode of action is based on the premature termination of viral nucleic acid synthesis. The intracellular phosphorylation of the nucleoside analog produces 5′-triphosphate metabolites, which are competitively incorporated into viral RNA nascent chains. This leads to the formation of incomplete viral RNA chains [56]. More than 25 nucleoside analogs have been approved for use as antiviral drugs against hepatitis [57,58], herpesvirus [59], and HIV infections [60].

An adenosine analog, BCX4430 (Galidesivir) is designed to inhibit viral RNA polymerase indirectly through nonobligate RNA chain termination. In an isolated enzyme transcription assay, BCX44330-triphosphate suppressed HCV NS5B RNA polymerase activity. While in a template-directed primer extension assay, it induced premature termination of RNA chain synthesis by HCV RNA [61]. It has exhibited antiviral efficacy against different flaviviruses (WNV, TBEV, and ZIKV) both in vitro and in vivo [62–64]. BCX4430 significantly reduced the viral CPE of different strains of ZIKV in various cells. Treatment of ZIKV-infected AG129 mice with BCX4430 at 300 mg/kg BID for 8 days via the intramuscular (i.m.) route starting 4 hours prior to infection resulted in a significant reduction of viremia and improved survival up to 87.5%. The protective effect of BCX4430 was also seen when treatment was started 1 day pi, and there were significant delays in mortality when treatment was delayed by up to 5 days pi [64].

NITD008, another nucleoside analog that targets RdRp, was originally designed for the treatment of DENV. In a primer extension-based RdRp assay, a triphosphate derivative of NITD008 reduced the levels of RNA products [65]. The compound significantly reduced different strains of ZIKV titters in vitro. In an A129 mouse model, administration of the drug at 50 mg/kg QD for 4 days via p.o. significantly reduced peak viremia and protected 50% of the mice from death and prevented neurological symptoms, while mice in the control group had a 100% mortality rate [66]. NITD008 was also evaluated against DENV. In AG129 mice, p.o. administration of the compound at 25 mg/kg BID for 3 days starting from day 0 pi resulted in a significant reduction of viremia. However, it provided no benefit when treatment started at day 3 pi [67]. Notably, NITD008 was not advanced to clinics due to its preclinical toxicity. However, this drug could be safe when used for a short treatment period for acute diseases such as dengue fever. In line with this, the drug did not exhibit any side effects during a 3-day treatment in the efficacy studies in an animal model [65].

An existing nucleoside analog, 7-deaza-2’-C-methyladenosine (7DMA), was initially developed for the treatment of HCV. 7DMA significantly reduced ZIKV RNA levels and almost completely inhibited the expression of viral antigen in Vero cells. The compound retained its antiviral potency even when the treatment was delayed until 10 hours pi. Administration of 7DMA at 50 mg/kg QD via p.o. exhibited a significant reduction in viremia and delayed progression of disease in ZIKV-infected AG129 mice [68]. 7DMA also significantly reduced WNV titers and suppressed the expression of WNV surface E antigen in nm or low μM concentrations in vitro. In BALB/c mice, i.p. administration of 7DMA at 25 mg/kg BID at time of infection and day 1 pi resulted in 100% survival of WNV-infected mice. When administered on day 3 pi, the compound was still highly effective, resulting in a 90% survival rate. However, the antiviral effect of 7DMA significantly dropped when the treatment was started at day 8 pi [69].

Compound 17, a cyclic phosphoramidate prodrug of 2′-Deoxy-2′-Fluoro-2′-C-Methylguanosine, was found to be effective against all DENV serotypes. Compound 17 showed high levels of triphosphate in peripheral blood mononuclear cells (PBMCs) after oral dosing in several animal species, including mice, dogs, and monkeys. In an AG129 mouse model, p.o. administration of compound 17 at 100 and 300 mg/kg BID for 3 days immediately after infection reduced viremia by 1.6 and 2.2 logs, respectively. However, in dogs, the compound caused mild pulmonary inflammation and hemmorrhage, and the “no observed adverse effect level” (NOAEL) could not be achieved [70]. Due to the severity observed in the preclinical safety assessment, the compound did not progress to the clinical stage. Nevertheless, the findings of this study demonstrated the potential of monophosphate prodrugs for DENV infection. In addition, a suitable prodrug moiety was identified to effectively deliver the monophosphate into PBMCs, one of the major DENV replication sites [71].

A study evaluated the antiviral potential of AT-752, an oral prodrug of a guanosine nucleotide analog against flaviviruses. AT-281, the free base of AT-752, demonstrated significant antiviral activity against DENV2 and DENV3, and other flaviviruses, including JEV, WNV, YFV, and ZIKV in vitro [72]. In vivo antiviral activity of AT-752 was evaluated in DENV2-infected AG129 mice via p.o. at 1,000 mg/kg, 4 hours viral prior to infection and BID at 500 mg/kg after 1 hour pi for 7 days. Treatment with AT-752 resulted in a significant reduction of viremia and improved the survival of the infected mice compared to the mice in the control group. Substantial levels of the active triphosphate metabolite AT-9010 were detected in PBMCs of mice, rats, and monkeys following p.o. administration of AT-572 [72]. The study demonstrated the antiviral mechanism of AT-572 is chain termination of RNA synthesis. In the RNA template-primer elongation assay with DENV2 RNA polymerase, AT-9010 competed with GTP leading to termination of RNA synthesis [72]. The hemisulfate salt AT-527 of AT-511, which is a diastereomer of AT-281, is currently under clinical development for HCV infection [73], and it is also being evaluated as a potential treatment option for COVID-19 [74].

A nonnucleoside compound, 3-chloro-N-[(amino)carbonothioyl]-1-benzothiophene-2-carboxamide (TPB), was identified as a potential antiviral agent against ZIKV using a structure-based approach. Molecular docking analysis revealed that TPB interacts with residues in the target site of the ZIKV RdRp forming 3 hydrogen bonds in direct contact with 2 aspartic acid residues in RdRp. TPB strongly inhibited ZIKV growth by >99% as determined by genome copies and infectious virus in the supernatants. In immunocompetent BALB/c mice, i.p. administration of TPB at 25 mg/kg Q12h for 3 times prior to infection reduced levels of ZIKV RNA to nearly 40-fold lower as compared to the control group [75].

Sofosbuvir, an existing FDA-approved HCV drug, was found to be effective against ZIKV. The drug inhibits HCV replication by inhibiting its RNA polymerase. The active sites of the RNA polymerases of HCV and ZIKV share close structural similarities, with all the residues contacting sofosbuvir conserved between HCV NS5B and ZIKV NS5. Hence, sofosbuvir could interact with ZIKV NS5 and inhibit its RNA polymerase activity. In vitro evaluation of the drug revealed its ability to block replication of different ZIKV strains in different cells [76,77]. Sofosbuvir inhibited ZIKV replication, reduced apoptosis, and restored the antiviral immune response in hNPCs. In addition, sofosbuvir protected ZIKV-infected neurospheres from cell death and retained their size. Sofosbuvir was evaluated in 5-week-old wild-type (WT) C57BL/6 mice treated with anti-IFNα receptor 1 (Ifnar1) antibody and infected with a mouse-adapted African ZIKV strain (Dakar 41519). Administration of sofosbuvir via drinking water at 33 mg/kg QD for 7 days starting from day 1 pi prevented weight loss and death in 50% of treated mice [76]. In another study, i.p. or p.o. administration of sofosbuvir at 50 mg/kg, QD for 10 days in ZIKV-infected adult nonobese diabetic severe combined immunodeficiency (NOD/SCID) mice significantly reduced viremia [77]. The dosage was calculated based on the animal equivalent dose (AED) from the recommended 400 mg/kg/day for the treatment of HCV infection in humans. Despite the fact that the dose was higher by weight than the human dose (400 mg), species scaling by body surface area corresponds to a scaled human equivalent dose (HED) of 4 mg/kg, which is less than the human sofosbuvir dose for a 70-kg individual. The calculation also accounts for the fact that larger animals have a lower metabolic rate and thus require a lower drug dose per kg of body weight [77]. Administration of sofosbuvir (44 or 440 mg/kg/day) via p.o. for 14 days did not cause liver toxicity in mice with humanized livers [78]. In addition, it was demonstrated that sofosbuvir treatment was well tolerated in pregnant mice, and there was no detectable ZIKV amplification in the treated fetuses [77]. Moreover, the drug has been shown to penetrate the brain and placenta [79]. The modest efficacy of sofosbuvir in mice could be associated with its low stability in rodent serum. Therefore, its efficacy against ZIKV should be assessed in nonhuman primates (NHPs). Furthermore, as sofosbuvir is used in combination with ledipasvir for the treatment of HCV infection, its combination therapies with other drugs may yield improved efficacy against ZIKV and other flaviviruses.

### Host-targeting antivirals

#### Entry inhibitors

Prochlorperazine (PCZ) is a dopamine D2 receptor (D2R) inhibitor and has been clinically approved to treat headaches, vomiting, and nausea. The compound suppressed infection of DENV and JEV in vitro. PCZ pretreated cells exhibited an antiviral effect in cells expressing D2R but not in D2R knockdown shD2R-N18 cells. This finding indicates that the antiviral effect of PCZ is dependent on D2R. In addition, PCZ treatment disrupted clathrin distribution, which inhibits DENV entry via clathrin-mediated endocytosis. In DENV-infected signal transducers and activators of transcription 1 (STAT1)-deficient (Stat1^−/−)^ mice, immediate treatment with PCZ completely protected the infected mice against death. When treatment started 6 hours pi, PCZ delayed deaths and improved the survival of the infected mice [80].

Approved anticancer drugs, sunitinib/erlotinib have exhibited antiviral activity against DENV, ZIKV, and WNV in vitro. Sunitinib/erlotinib inhibit viral entry and production of infectious virus particles by targeting host cell kinases AP2-associated protein kinase 1 (AAK1) and cyclin G-associated kinase (GAK), which regulate adaptor protein-mediated intracellular membrane trafficking [81]. In murine models (AGB6 and AG129), QD or BID treatment with a combination of sunitinib/erlotinib (30 to 60 mg/kg) for 5 days starting from 0-hour DENV infection significantly reduced viral load, morbidity and mortality, and altered host cytokine responses. The reduction in the viral load was more prominent when the drug was administered twice daily as a result of the maintenance of a higher serum drug concentration [81,82]. Importantly, the doses used for each drug were near the equivalent of the approved human dose calculated based on the body surface area. These doses were also confirmed to be nontoxic in the murine models of dengue [81]. Another study looked into whether the protection provided by sunitinib/erlotinib in the DENV-infected murine model lasts longer. Five days of treatment with sunitinib/erlotinib protected 70% of the mice until day 14. However, between 14 and 25 days after treatment, the surviving mice developed paralysis as treatment did not prevent DENV replication in the brain likely due to the poor blood–brain barrier (BBB) permeability of sunitinib and erlotinib [82].

#### Fusion inhibitors

The 25-hydroxycholesterol (25HC) is an oxysterol synthesized from cholesterol by cholesterol-25-hydroxylase (CH25H). The compound exhibited in vitro antiviral activity against ZIKV, DENV, YFV, and WNV by blocking internalization during viral entry. The 25HC suppressed fusion between viral and cell membranes through modulation of lipid metabolism without affecting the virus–host cell binding. Treatment of ZIKV-infected BALB/C mice with 25 HC at 50 mg/kg QD for 7 days via i.p. significantly reduced viremia, In A129 mice, a similar treatment regimen with 25HC significantly reduced viremia and mortality, and surviving mice did not develop any clinical symptoms, while mice in the vehicle-treated group died within 14 days with severe neurological symptoms, including paralysis and hind limb weakness. Treatment also reduced viral titers in the brains of ZIKV-infected mice. Its antiviral efficacy was also evident in an NHP model, in which treatment with 25HC (1.5 mg/kg QD for 7 days via i.v.) pre- and post-ZIKV infection shortened the duration of viremia and viral shedding in the urine and prevented fever in monkeys. The study also investigated whether 25HC could protect against ZIKV-associated neurological damage. Pretreatment with 25HC significantly suppressed ZIKV infection in the human cortical organoids. Notably, treatment of pregnant mice with 50 mg/kg of 25HC via i.p. prevented ZIKV infection in the fetal brain and also reduced the incidence of microcephaly [83].

Chloroquine is an antimalarial drug whose repurposing has been explored against a wide range of viruses [84,85]. Its antiviral efficacy against DENV was evaluated in an NHP model under prophylactic and therapeutic conditions. A reduction in the time of viremia and the levels of interferon gamma (IFNγ) and tumor necrosis factor alpha (TNFα) were observed in the treated group compared to the control group [86]. Chloroquine exhibited antiviral activity against different strains of ZIKV in different cells and mouse neurospheres [87]. The compound has been shown to inhibit ZIKV infection by suppressing its internalization without affecting virus binding to cells or RNA synthesis [88]. It also affected the later stages of ZIKV replication. Its mechanism is associated with the prevention of virus–endosome fusion through inhibition of acidification of the endosomes [87]. The in vivo antiviral efficacy of chloroquine was investigated in BALB/c and A129 mice. Treatment of infected mice with 100 mg/kg QD for 5 days via the intragastric (i.g.) route starting from 6 hours prior to infection significantly reduced viremia. Treatment of ZIKV-infected pregnant ICR mice with chloroquine (100 mg/kg) reduced ZIKV infection and apoptosis in the embryonic brains and protected them from microcephaly [88]. Chloroquine has been shown to be safe for pregnant women and infants. Two clinical trials have been conducted for chloroquine for DENV infection. In the trials, chloroquine did not reduce the duration of viremia and NS1 antigenemia, but demonstrated a reduction in fever clearance time with a lower incidence of dengue hemorrhagic fever (DHF) [89] and decreased the intensity of pain in the patients and improved their ability to perform daily activities [90].

#### Replication inhibitors

AR-12 is a celecoxib-derived FDA-approved investigational new drug (IND) drug for cancer treatment [91]. The compound suppressed the replication of all DENV serotypes in vitro and downregulated the phosphatidylinositol-3-kinase (PI3)/protein kinase B (AKT) activity and expression of 78-kDa glucose-regulated protein (GRP78) in DENV-infected cells. Treatment of DENV-infected ICR suckling mice with a single dose of AR-12 (25 mg/kg, i.p. and i.c.) reduced the expression of DENV nonstructural proteins and the production of infectious viral particles, neuropathological symptoms and mortality. The antiviral efficacy of AR-12 was still retained when treatment was initiated 12 hours and 24 hours pi [92]. A recent study reported the potency of an AR-12 derivative, P12-34 with enhanced antiviral activity against DENV, ZIKV, and JEV. The compound also improved the survival and symptoms in a DENV-infected Stat1^−/−^ mouse model at 2.5 mg/kg QD for 5 days administered via i.p. It was found that AR-12 and P12-34 inhibit viral replication by targeting dihydroorotate dehydrogenase (DHODH) [93].

Ivermectin, a drug that has been used for over 30 years for the treatment of parasitic infections in humans, has been shown to be effective against multiple flaviviruses. In a recent study, the treatment of ZIKV-infected cells with ivermectin reduced levels of NS5 in the nucleus [94]. A few studies propose that this activity is likely associated with inhibition of host importin, IMPα/β1 by ivermectin, which subsequently blocks the nuclear trafficking of viral proteins [94–96]. A bimolecular fluorescence complementation (BiFc) analysis showed inhibition of the DENV NS5–IMPα interaction by ivermectin [96]. Ivermectin was found to significantly inhibit ZIKV infection in different cells [31]. However, it failed to improve the mortality and morbidity of infected Ifnar1 knockout mice (Ifnar1^−/−/^) [97]. The drug was unsuccessful in the Phase III clinical trial. It has been proposed that changing the dosage regimen may improve the efficacy of ivermectin [98]. Currently, there is an ongoing clinical trial for the evaluation of ivermectin for the treatment of dengue (NCT02045069), and another clinical trial looking into the pharmacokinetics and pharmacodynamics of ivermectin in pediatric dengue patients has been completed (NCT03432442) (www.clinicaltrials.gov).

#### Assembly and egress inhibitors

Many studies have explored the potential antiviral activity of iminosugars against DENV. Iminosugars inhibit ER α-glucosidase I and II enzymes, causing improper glycosylation and misfolding of viral glycoproteins. It has been demonstrated that they reduce the formation and stability of prME heterodimer [99,100]. One of the most advanced iminosugars for dengue is celgosivir, which was initially developed for the treatment of HCV. Multiple in vivo studies have been conducted to optimize the ideal treatment regimen for DENV infection. In AG129 mice, celgosivir treatment at 10 and 50 mg/kg BID via p.o. induced a significant reduction in viremia when administered at the time of infection. The study also investigated the response of celgosivir when treatment was begun on day 3 pi to mimic dengue fever patients, who usually present at a clinic when the viremia is at its peak. However, celgosivir did not induce a significant reduction in the viremia when treatment started at the time of peak viremia compared to the 9-fold reduction of peak viremia observed when treatment started at the time of infection. As it has been previously found that increasing the frequency of dosing is more effective than a single dose, the effect of QID of celgosivir was investigated. QID of celgosivir at 50 mg/kg via p.o. starting from day 2 and 3 pi significantly reduced the level of viremia [67]. These findings strongly support the importance of a dosing regimen for DENV infection, and a similar regimen could be effective in a human clinical trial.

A novel iminosugar termed UV-12 inhibited DENV in vitro. UV-12 demonstrated inhibitory activity against both α-glucosidase I and II in a purified enzyme assay as well as inhibition of ER α-glucosidase enzymes in a free oligosaccharide (FOS) assay. In an ADE AG129 mouse model, treatment with the compound (20 and 100 mg/kg TID for 7 days via p.o.) 1 hour before lethal DENV infection resulted in 100% survival of mice. Treatment resulted in a significant reduction in viral load in the kidney and small intestine but not in the serum, liver, and spleen and reduced the levels of several circulating cytokines and chemokines [101].

Iminosugar UV-4B inhibited purified ER-glucosidases I and II and reduced infectious virus titer and RNA of all 4 DENV serotypes in cell culture. In a lethal ADE DENV2 mouse model, p.o. administration of UV-4B at 10 to 100 mg/kg TID for 7 days exhibited protection even when treatment started 48 hours pi [100].

A study has explored the antiviral potential of an acetyl-Coenzyme A carboxylase (ACC) inhibitor, PF-05175157. PF-05175157 inhibited both ACC1 and ACC2, and lipidomics analyses revealed that the compound significantly modifies host lipid metabolism, which impairs virus biogenesis by affecting replication and particle morphogenesis. The compound significantly reduced the virus yield of DENV, ZIKV, and WNV in vitro. Treatment of WNV-infected mice at 20 mg/kg BID for 7 days via p.o. starting from 1 day before infection did not completely control virus infection but significantly reduced viral load in serum and kidneys of infected mice. Evaluation of its potency in ACC2-deficient (ACC2^-/-^) mice resulted in a significant reduction in the viral load in serum but not in the kidney, indicating ACC1 is also necessary to achieve the effect seen with PF-05175157 [102].

### Unknown mechanisms

Memantine is an FDA-approved N-methyl-D-aspartate receptor (NMDAR) antagonistic drug used for the treatment of Alzheimer disease and is known for its neuroprotective properties. NMDARs contribute to the neuronal damage induced by ZIKV infection. The effects of memantine were tested in ZIKV-infected primary cultured neurons, and it was found that the drug prevented neuronal cell death by binding to NMDAR. It also reduced neurodegeneration and microgliosis in the brains of infected mice [103].

### Multiple-target antivirals

Niclosamide is an FDA-approved anthelmintic drug. Through drug repurposing screening, niclosamide was found to be effective against flaviviruses including DENV, WNV, JEV, and YFV [32]. Treatment of ZIKV-infected cells with niclosamide inhibited virus production, reduced certain inflammatory mediators, and prevented cell apoptosis. In a humanized ZIKV-infected chick embryo model, the drug improved survival rate, partially rescued ZIKV-induced microcephaly and reduced infection of hiNSCs [104]. Cotreatment of DENV-infected ICR suckling mice with a single dose of the drug (2 and 5 mg/kg via i.p.) only partly reduced DENV replication, DENV-induced acute viral encephalitis-like symptoms, and mortality [105]. A study suggested that niclosamide blocks the formation of the NS2B-NS3 complex, which subsequently inhibits flavivirus polyprotein processing [32]. Recently, it was shown that the mechanism is associated with endosomal deacidification, which blocks fusion of the viral E protein with the host membrane [105].

Ribavirin is a synthetic nucleoside analog and it is an FDA-approved drug with broad-spectrum antiviral activity and has been widely used for the treatment of chronic hepatitis B virus (HBV) and HCV. Ribavirin has been shown to suppress ZIKV replication in cells and prevent ZIKV-induced cell death. In ZIKV-infected Stat1^−/−^, ribavirin treatment at 15 mg QD for 3 days via i.p. significantly suppressed viremia on day 2 and 3 pi. However, at 2 days after discontinuation of ribavirin, the reduction in viremia was insignificant compared to the untreated mice in the control group. Ribavirin also did not suppress the viral load in the brains on day 3 and 5 pi. In terms of survival, treatment with ribavirin (15 mg) for the first 3 days after infection did not prolong the survival of infected mice. On the contrary, daily administration of 10 mg of ribavirin throughout the course of infection successfully prolonged the survival of the infected mice throughout the course of infection. However, daily administration of a lower concentration of ribavirin (2 mg) did not prolong the survival of ZIKV-infected mice [106]. Similarly, for DENV infection, lower concentrations of approximately 1 to 2 mg of ribavirin did not reduce viremia in AG129 mice [107].

A drug repurposing screen identified emetine as a potential antiviral drug for ZIKV. Emetine is an FDA-approved compound for amoebiasis and has demonstrated broad-spectrum antiviral activity [108]. This compound suppressed the replication of different strains of ZIKV in multiple cells. Its in vivo antiviral efficacy was evaluated in 2 mouse models. Treatment of ZIKV-infected immunocompetent SJL mice with emetine at 1 mg/kg QD for 6 days via i.p. reduced approximately 10-fold of circulating ZIKV. In ZIKV-infected Ifnar1^−/−^ mice, treatment with emetine at 1 and 2 mg/kg QD for 3 days via i.p. starting 1 day before infection significantly reduced serum viral load. In addition, treatment with emetine both pre- and post-ZIKV infection significantly reduced the levels of NS1 protein and ZIKV RNA in serum and liver. A cellular thermal shift assay (CETSA) demonstrated that emetine bound directly to ZIKV NS5. In addition to that, emetine was found to directly inhibit ZIKV NS5 RNA polymerase in a cell-free ZIKV NS5 RNA polymerase. Emetine binding interactions with ZIKV NS5 polymerase were further confirmed through molecular modeling and docking studies. The study also demonstrated that emetine accumulated in lysosomes and disrupted lysosomal function, resulting in the inhibition of autophagy. This may subsequently interfere with the autophagy-dependent virus infection and cellular trafficking of lipids required for virus particle assembly [109].

Lycorine is a benzyl phenethylamine alkaloid with diverse biological activity and has also demonstrated broad-spectrum antiviral activity. Treatment of ZIKV-infected cells resulted in a reduction of RNA synthesis and ZIKV protein. A CETSA demonstrated direct binding of lycorine to ZIKV NS5. In vitro RNA polymerase assays further showed that lycorine inhibited RdRp activity. In addition to these assays, molecular modeling and docking studies supported binding interactions of lycorine with finger domains of ZIKV RdRp. In previous studies, HSP70 [110] and 2A protease [111] were proposed to be the targets for the antiviral activity of lycorine against HCV and enterovirus A71 (EV71A), respectively. However, Chen and colleagues found that the binding energy of lycorine with HSP70 and ZIKV NS3 was weaker compared to the positive control. Treatment with lycorine at 5 mg/kg and 10 mg/kg QD for 14 days via i.g. improved the survival of ZIKV-infected AG6 mice by 66% and 83%, respectively. Treatment with lycorine also resulted in a significant reduction of ZIKV viral RNA in the serum, brain, and liver as well as a reduction in the inflammatory response in the infected mice [112].

HTS of the FDA-approved drug library for JEV inhibitors identified manidipine as a potential candidate. Manidipine is a calcium channel inhibitor that has been approved for the treatment of hypertension. The compound demonstrated significant inhibition of viral replication of JEV, ZIKV, DENV, and WNV in vitro. Treatment of JEV-infected BALB/c mice with manidipine at 25 mg/kg for 21 days via i.p. significantly reduced the viral load and alleviated damage in the brain. However, the drug did not reduce the level of viral RNA in the serum or spleen. These findings suggest that manidipine protected the mice from JEV-induced lethality by lowering the viral load in their brains. Adaptive mutant analysis revealed that a single mutation in the transmembrane domain of NS4B conferred resistance to manidipine, suggesting NS4B as the potential target of the compound [113].

Hydroxychloroquine (HCQ) is a derivative of chloroquine. It is an FDA-approved class C drug that is used to treat pregnant women with autoimmune diseases and malaria. The compound has significantly reduced ZIKV titers in placental trophoblast cells [114]. HCQ-treatment (40 mg/kg QD for 5 days via i.p.) of ZIKV-infected pregnant WT mice beginning at day 1 pi, significantly reduced ZIKV RNA in the placentas, decreased placental damage, rescued placental insufficiency, and subsequently reduced ZIKV infection in the fetal brain. Interestingly, the reduction of ZIKV by HCQ in the pregnant mice was specific to the placentas as treatment did not reduce ZIKV titer in the serum, spleen, or maternal decidua. Using ZIKV-infected Atg16l1HM mice (autophagy gene, Atg16l1-deficient mice), it was further confirmed that the activity induced by HCQ is associated with inhibition of autophagy [115]. HCQ has also been found to inhibit NS2N-NS3 protease activity, and molecular docking further supported the binding of HCQ to the NS2B-NS3 protease active site [114].

### Unknown targets

Hippeastrine hydrobromide (HH) is a chemical analog of a natural compound that has shown antiviral activity against avian influenza (H5N1) and HCV. HTS identified its potency against ZIKV. The compound significantly suppressed the production of viral RNA and infectious particles in ZIKV-infected hNPCs. Furthermore, it rescued microcephaly-related effects in human fetal-like forebrain organoid cultures. Prophylactic treatment of SCID-beige mice with HH (100 mg/kg QD via s.c.) 12 hours prior to ZIKV infection significantly reduced ZIKV RNA in the brain and ZIKV-induced cellular apoptosis. Administration of HH at 24 hours and day 5 pi also significantly decreased levels of ZIKV RNA in the ZIKV-infected mouse brains [117].

An antimalarial drug, amodiaquine (AQ), suppressed ZIKV infection in hNPCs. In a SCID-beige mouse model, AQ at 40 mg/kg QD via i.p. significantly suppressed levels of ZIKV RNA and inhibited cellular apoptosis in the brain [117].

Azithromycin is a broad-spectrum macrolide antibiotic with antiviral potential [118]. It has been shown to reduce viral proliferation in glial cells [119]. In ZIKV-infected immunocompetent ICR suckling mice, azithromycin (1 or 10 mg/kg via i.p.) reduced viral titer, increased survival, decreased monocyte infiltration into the brain, and the mice displayed decreased signs of illness and neurological disease in a dose-dependent manner [120].

### Target product profile for potential flavivirus drugs

A target product profile (TPP) for drugs serves as guidance to design a product and outline appropriate preclinical and clinical testing. Desirable properties of an antiviral candidate include low cost, ease of administration, and a favorable risk–benefit profile. For antiviral drugs against DENV, ZIKV, and other flaviviruses, the target populations are adults and children, including infants and pregnant women presenting clinical symptoms [121]. The drugs can also be used as short-term prophylaxis for travelers to endemic regions. For the treatment of pregnant women infected with ZIKV, the drugs should be able to penetrate the BBB, placental barrier, and must have a high level of safety. Ideally, oral administration is preferred for flavivirus therapeutics due to its convenience and increased patient compliance. Two or 3 times doses per day may be necessary to maintain the effective concentrations of the drug. DENV drugs should ideally act on all DENV serotypes. In terms of efficacy, the drugs should eliminate the circulating virus from the bloodstream. For ZIKV, the drugs should be able to reduce the risk of neurological impairment of the fetus.

### Limitations and challenges in the antiviral drug development for flaviviruses

For some of the drugs that are listed in this review, the targets or mechanisms were solely predicted based on molecular docking, which very often provides results that do not correspond with the experimental binding affinities due to limited sampling of both ligand and receptor conformation in pose prediction and the use of approximated scoring functions [122,123]. Therefore, the molecular docking findings should be backed by X-ray crystallography, resistance selection, and reverse genetics studies. X-ray crystallography has been used successfully to identify and validate the binding interactions between different HIV-1 drugs and viral proteins such as proteases [124] and reverse transcriptase [125]. In recent years, advancements in cryo-EM have led to the determination of the structures of protein complexes of different sizes at near-atomic resolution. This enables us to deduce the detailed interaction between the drugs and their protein targets, providing insights into the molecular mechanisms. In contrast to X-ray crystallography, which requires crystallized protein samples, cryo-EM allows structural determination of proteins in their native state [126,127]. Recently, cryo-EM was used to establish the mechanism of remdesivir-induced SARS-CoV-2 RdRp stalling [128].

In relation to drug repurposing, a study by Tummino and colleagues found that most of the repurposed drugs tested for SARS-CoV-2 are cationic amphiphilic drugs (CADs), and they exhibited antiviral activity in cells by inducing phospholipidosis rather than specific on-target activity [129]. Drug-induced phospholipidosis is likely to occur as a result of the direct interaction of CADs with membrane phospholipids, which subsequently interferes with viral replication. The study also demonstrated that the phospholipidosis-inducing drugs did not induce efficacy in vivo, even when tested at high concentrations for an extended period [129]. In contrast, the drugs discussed in our review have shown potent in vivo efficacy. However, it is important to rule out drug-induced phospholipidosis prior to clinical investigations.

Several studies have shown a positive correlation between viremia and disease severity [130–132]. Therefore, early treatment within 48 to 72 hours of fever onset with antiviral drugs has been proposed to be ideal for reducing the viral load and, subsequently, the disease severity. Furthermore, in in vivo antiviral efficacy studies, most of the drugs were demonstrated to be more effective when treatment was started before or at 0 hour virus infection and became less efficacious once viremia reached the peak [67]. However, in most instances, patients with fever take a wait-and-see approach with self-medication and delay seeing a physician until their symptoms worsen. By the time they seek medical attention, the peak stage of viremia would have passed. As a result, using the reduction in peak viremia level as a clinical endpoint for antiviral treatment is difficult.

One of the challenges of animal models for flaviviruses has been the ability to mimic human disease. AG129 mice have been widely used for the evaluation of the efficacy of antiviral agents. Nevertheless, the AG129 model has several disadvantages, such as dependence on highly mouse-adapted strains and the lack of clinical symptoms (fever or rashes) seen in humans. Furthermore, the AG129 mice die of DHF/dengue shock syndrome (DSS)-like diseases after 1 to 2 days of peak viremia. This makes it challenging to evaluate the effect of the antiviral drugs on reducing viremia and its correlation to disease severity [67]. In this context, including a nonlethal viremia model in preclinical efficacy testing could give a clearer picture of the kinetics of viremia clearance following treatment with antivirals.

The development of resistance is a major challenge for antiviral drugs. In line with this, a combination of 2 antivirals with distinct modes of action may be required to minimize resistance and also, in the case of DENV, to inhibit all serotypes. Many approved treatment regimens for HIV and HCV are based on combinational therapies. Combination therapies have shown enhanced antiviral efficacy and improved clinical outcomes [133]. Another challenge is toxicity observed in humans during clinical trials, despite safety observed in preclinical evaluation. About 89% of novel drugs fail human clinical trials, with toxicity in humans accounting for half of those failures [134].

### Lessons from the past and now

In the past years, clinical trials have been conducted for dengue using repurposed drugs: celgosivir [135], balapiravir [136], chloroquine [89], prednisolone [137], and lovastatin [138]. Despite demonstrating a good safety profile in acute dengue patients, these drugs failed to meet the prespecified trial endpoints, including time to resolution of viremia and serum NS1. In addition to the clinical endpoints, the dosing regimen also plays a critical role. For example, in the case of celgosivir, despite its strong antiviral efficacy in preclinical studies, the drug failed to reduce viremia and fever in patients in a clinical trial. In the clinical trial, a 400 mg loading dose followed by 200 mg every 12 hours was used. This dose was based on the 50 mg/kg used in the preclinical studies and translated to HED. A recent study demonstrated that increasing the frequency of treatment with celgosivir to 4 times daily yielded a significant reduction in the levels or viremia even when treatment started on day 2 or 3 pi [67]. Based on these findings, a Phase II clinical trial with increased frequency of dosing was approved (NCT02569827) to assess the efficacy of celgosivir in adult dengue patients using a new dose regimen. However, the Phase II clinical trial for celgosivir has been withdrawn due to a lack of VC funding (www.clinicaltrials.gov).

Chen and colleagues attempted to understand why balapiravir failed in a clinical trial for the treatment of DENV infection. Balapiravir is a cytidine analog prodrug (R1479), a DENV polymerase inhibitor. Since, DENV infection had already been well established by the time patients were treated with balapiravir, the EC_50_ obtained for delayed treatment should reflect the therapeutic condition. In line with this, the study found that when treatment started 24 hours pi, the EC_50_ increased to 12.85 μM compared to immediate treatment after infection [139]. The study also found that the expression of cytokines by PBMCs during DENV infection reduced the conversion of the prodrug to its active form, resulting in a reduction in antiviral activity. The drug, despite demonstrating a good pharmacokinetics profile, only marginally reduced the viremia in DENV2-infected AG129 mice, even at a concentration as high as 100 mg/kg administered immediately after infection. The study also indicated that balapiravir had low efficacy on DENV-infected hepatocytes, which could contribute to its lack of efficacy in dengue patients as hepatocytes are one of the major replication sites for DENV [139].

Numerous clinical trials have been conducted to evaluate the efficacy of repurposed drugs for the treatment of SARS-CoV-2, but none so far has shown a promising impact on COVD-19, particularly in large clinical trials. Remdesivir had no effect on mortality in randomized controlled trials (RCTs) [140]. Yan and colleagues suggested that the preclinical assumptions on remdesivir overestimated its efficacy and undermined its toxicity in humans. The study pointed out that the in vitro analyses overlooked some of the key considerations for prodrugs (i) short t1/2 in vivo; and (ii) cytotoxicity in hepatocytes. In the in vivo context, the higher hepatotoxicity of remdesivir, a McGuigan prodrug in humans, compared to NHPCs due to species differences in hepatic extraction was overlooked [141]. Sofosbuvir, another McGuigan prodrug, was also found to have higher hepatic extraction in humans when compared to NHPs [142]. Furthermore, the dosing used in humans was lower than the HED of NHP, but up-dosing for improved efficacy is hampered by nephro- and hepatotoxicity [140,141,143]. If these limitations were carefully accessed, the poor efficacy during the clinical trial would have been predicted.

In double-blind RCTs for ivermectin, it was found that the drug did not reduce the proportion of hospitalized patients with severe COVID-19 [144]. In another RCT involving patients with nonsevere COVID-19 and no risk factors, ivermectin did not have any effect on the proportion of PCR positives [145]. For HCQ, large observational clinical trials and RCTs have demonstrated no effect in reducing mortality or mobility. It is important to note that in a RCT, a high-dose of chloroquine (parent compound of HC) exhibited cardiac toxicity and higher fatality rates in COVD-19 patients [146]. Other repurposed drugs like favipiravir did not improve viral clearance [147], while lopinavir-ritonavir did not show clinical benefits against SARS-CoV-2 [148]. RCTs on dexamethasone (an immunomodulator) showed a significant increase in survival and morbidity in patients with moderate or severe COVID-19 [149,150]. These results emphasize the importance of adequately powered and well-designed clinical trials for repurposed drugs in the treatment of viral diseases. Furthermore, they highlight that the in vitro antiviral potency should be taken cautiously, and, importantly, the virus target specificity should be adequately characterized.

To date, the HIV-1 therapy pipeline has the most successful story in treating any single human infectious disease with more than 30 FDA-approved drugs. These drugs generally fall into classes based on their molecular mechanisms and drug resistance profiles. The antiretroviral classes include nucleoside reverse transcriptase inhibitors, nonnucleoside reverse transcriptase inhibitors, protease inhibitors, integrase inhibitors, CCR5 antagonists, attachment inhibitors, and postattachment inhibitors [151–153]. Unlike most of the repurposed drugs, which have diffuse viral targets, antiretroviral target specificity has been well characterized through in vitro or in vivo selection of HIV-resistant variants. Importantly, the success of HIV-1 antiretroviral drug therapy is partly contributed by drug regimens that use combinations of agents directed against at least 2 distinct molecular targets, which has improved the overall efficacy and durability of therapy [151–153]. In the case of HCV, the development of direct-acting antivirals and the usage of combination drugs has contributed to the global eradication of HCV [154,155].

## Concluding remarks and future perspectives

This review demonstrates that from 2015 to 2021, about 37 compounds have been evaluated for their efficacy against flaviviruses in vivo, 20 of them are repurposed drugs and most of them possess broad-spectrum antiviral activity. This review also suggests that the E protein, NS5 RdRp, NS2B-NS3 protease, and ER α-glucosidase, are the dominant targets for drug development against flaviviruses. Also, the compounds targeting host proteins are only focused on general targets rather than specific targets for flavivirus pathogenesis. Therefore, a few approaches have to be taken in the quest for the identification of potent antiviral drugs against flaviviruses: (1) screening of more libraries of FDA-approved drugs, compounds in clinical trials, and preclinical candidates through better integrative platforms; (2) exploring the potential of other viral proteins than E, RdRp, and NS2B-NS3; (3) building libraries of druggable host proteins identified through PPI networks; (4) developing derivatives of existing potent compounds to improve efficacy and safety; and (5) focusing on developing multitarget antivirals and/or combination drug. In conclusion, integrative drug repurposing strategy holds the potential for the identification and development of an effective antiviral drug against flaviviruses with lower costs and shorter timelines. For increased success and improved efficacy, this strategy should be used along with the drug combination approach. The development of a broad-spectrum antiviral drug would be particularly ideal to tackle the large number of viruses in the flavivirus genus.

Even though most of the drugs discussed here exhibit strong antiviral potency against different flaviviruses, there are several concerns that need to be carefully addressed to ensure these drugs are worth clinical trial evaluation. Firstly, the specific target and the mechanism of action of the drugs need to be characterized. In vivo efficacy should mimic treatment in patients in terms of the dose and regimen. The cytotoxicity and antiviral potency in vitro ideally should be evaluated in different types of cells, and the preclinical pharmacokinetics and toxicity should be adequately investigated, taking into account species differences.

**Key Learning Points**

- The drug repurposing strategy has opened avenues for the rapid identification of effective antivirals. However, its preclinical efficacy and toxicity should be fully investigated.
- Drug-induced phospholipidosis should be evaluated for the candidate drugs.
- A number of repurposed drugs possess potential antiviral activity against flaviviruses.
- Most of the current antiviral compounds tested for flaviviruses target the envelope protein, NS5 RdRp, NS2B-NS3 protease, and ER α-glucosidase.
- The targets and mechanisms for most of the drugs tested against flaviviruses have not been adequately characterized. It is crucial to identify the specific drug target using reliable methods, such as X-ray crystallography and cryo-EM.
- Structural modification and changes in the dosage regimen of an existing drug could improve efficacy and safety.
- In vivo efficacy should mimic treatment in patients in terms of the dosing regimen and route of administration, in accordance with the target product profile (TPP).

**Top Five Papers**

1. Mercorelli B, Palu G, Loregian A. Drug Repurposing for Viral Infectious Diseases: How Far Are We? Trends Microbiol. 2018;26(10):865–76. Epub 2018 May 16. doi: 10.1016/j.tim.2018.04.004
2. Barrows NJ, Campos RK, Powell ST, Prasanth KR, Schott-Lerner G, Soto-Acosta R, et al. A Screen of FDA-Approved Drugs for Inhibitors of Zika Virus Infection. Cell Host Microbe. 2016;20(2):259–70. Epub 2016 Jul 28. doi: 10.1016/j.chom.2016.07.004
3. Tummino TA, Rezelj VV, Fischer B, Fischer A, O’Meara MJ, Monel B, et al. Drug-induced phospholipidosis confounds drug repurposing for SARS-CoV-2. Science. 2021;373(6554):541–7. Epub 2021 Jul 31. doi: 10.1126/science.abi4708. PubMed PMID: 34326236; PubMed Central PMCID: PMC8501941.
4. Watanabe S, Chan KW, Dow G, Ooi EE, Low JG, Vasudevan SG. Optimizing Celgosivir therapy in mouse models of dengue virus infection of serotypes 1 and 2: The search for a window for potential therapeutic efficacy. Antiviral Res. 2016;127:10–9. Epub 2016 Jan 23. doi: 10.1016/j.antiviral.2015.12.008. PubMed PMID: 26794905.
5. Yan VC, Muller FL. Why Remdesivir Failed: Preclinical Assumptions Overestimate the Clinical Efficacy of Remdesivir for COVID-19 and Ebola. Antimicrob Agents Chemother. 2021;65(10):e0111721. Epub 2021 Jul 3. doi: 10.1128/AAC.01117-21. PubMed PMID: 34252308; PubMed Central PMCID: PMC8448091.
